# Efficacy, Safety and Future Perspectives of JAK Inhibitors in the IBD Treatment

**DOI:** 10.3390/jcm10235660

**Published:** 2021-11-30

**Authors:** Patrycja Dudek, Adam Fabisiak, Hubert Zatorski, Ewa Malecka-Wojciesko, Renata Talar-Wojnarowska

**Affiliations:** 1 Department of Digestive Tract Diseases, Faculty of Medicine, Medical University of Lodz, 90-153 Lodz, Poland; adam.fabisiak@umed.lodz.pl (A.F.); hubert.zatorski@umed.lodz.pl (H.Z.); ewa.malecka-panas@umed.lodz.pl (E.M.-W.); renata.talar-wojnarowska@umed.lodz.pl (R.T.-W.); 2 Department of Biochemistry, Faculty of Medicine, Medical University of Lodz, 92-215 Lodz, Poland

**Keywords:** JAK inhibitors, small molecules, IBD, ulcerative colitis, Crohn’s disease

## Abstract

Although development of biologics has importantly improved the effectiveness in inducing and maintaining remission in inflammatory bowel disease (IBD), biologic therapies still have several limitations. Effective, low-cost drug therapy with good safety profile and compliance is therefore a substantial unmet medical need. A promising target for IBD treatment strategies are Janus kinase (JAK) inhibitors, which are small molecules that interact with cytokines implicated in pathogenesis of IBD. In contrast to monoclonal antibodies, which are able to block a single cytokine, JAK inhibitors have the potential to affect multiple cytokine-dependent immune pathways, which may improve the therapeutic response in some IBD patients. Tofacitinib, inhibiting signaling via different types of JAKs, has been already approved for ulcerative colitis, and several other small-molecule are still under investigation. However, one of the main concerns about using JAK inhibitors is the risk of thromboembolic events. Moreover, patients with COVID-19 appear to have an increased susceptibility for immunothrombosis. Therefore, thrombotic complications may become a serious limitation in the use of JAK inhibitors in the SARS-CoV-2 pandemic. As many questions about safety and efficacy of small molecules still remain unclear, in our review we present the current data regarding approved JAK inhibitors, as well as those in clinical development for the treatment of IBD.

## 1. Introduction

Inflammatory bowel disease (IBD), such as Crohn’s disease (CD) and ulcerative colitis (UC) are chronic, progressive, and inflammatory disorders, characterized by alternating episodes of relapse and remission, involving a lifelong treatment [1]. The etiology of IBD still remains poorly understood, although the current approach includes a combination of triggers, such as genetic susceptibility, environmental factors, or gut microflora [2]. A coincidence of these factors may lead to imbalance in the production of several pro-inflammatory and anti-inflammatory cytokines, which initiate an inappropriate mucosal immune response in the gastrointestinal (GI) tract [3,4]. Blocking the pathological pathways of production and release of pro-inflammatory cytokines has become the focus of current treatment of IBD. Conventional therapy of IBD includes aminosalicylates, corticosteroids, and immunosuppressive agents, such as azathioprine, mercaptopurine, or methotrexate. The introduction of antibody-based biologics, mainly tumor necrosis factor-α (TNF-α) inhibitors, has revolutionized the outcomes of IBD therapy, although many patients remain unresponsive or lose the response during treatment. The annual risk of loss of response to infliximab and adalimumab in patients with CD was reported to be 13% and 20%, respectively [5,6]. The cumulative relapse rates vary between 67% and 83% after 10 years [7]. Limitations of the biologics also include the problem of antidrug antibodies production and immunogenicity, which may also lead to the lack of an adequate response to the therapy [8]. Furthermore, the toxicity of anti-TNF-α medications cannot be neglected with pneumonia, tuberculosis, lymphoma, drug-induced skin lesions, and hepatotoxicity are regarded as the most common complications of the therapy. Therefore, there is a significant unmet medical need to search for drugs of another mechanism of action and maximize the treatment efficacy and compliance.

A promising target for new therapeutic IBD strategies are Janus kinase (JAK) inhibitors, which are small molecules that interact with many cytokines implicated in pathogenesis of IBD. JAKs are the enzymes which determine the signal transduction by interaction with signal transducers and activators of the transcription (STATs) pathway. Dysfunction and genetic variation of the JAK-STATs pathway has been associated, apart from IBD, with other various hematological and autoimmune diseases, such as myeloproliferative neoplasms, acute lymphoblastic leukemia, severe combined immunodeficiency disorders, multiple sclerosis, systemic lupus erythematosus, psoriasis, or rheumatoid arthritis (RA) [9]. JAK inhibitors have shown efficacy in IBD therapy in clinical trials and one of them, tofaticinib, has been already approved by the Food and Drug Administration (FDA) and European Medicines Agency (EMA) for the treatment of patients with moderate to severe UC [10].

Compared to antibody-based biologics, JAK inhibitors are small molecules, with the advantage of being the first targeted IBD therapy administered orally, which is more acceptable than the intravenous or subcutaneous route of administration. As JAK inhibitors can rapidly enter the systemic circulation, they are characterized by a rapid onset of action and may induce fast symptomatic relief [11]. In contrast to monoclonal antibodies, which are able to block a single cytokine (TNF-α, IL-12 or IL-23), JAK inhibitors have the potential to affect multiple cytokine-dependent immune pathways, which may improve the therapeutic response of some patients with IBD [12]. JAK-inhibitors influence the levels of a great number of interferons and interleukins. Among the latter, IL-6, IL-12 and IL-23 are important drivers of disease activity in IBD. Specifically, IL-6 is activated by JAK1, JAK2, and TYK2 via the STAT3 pathway, IL-23 via JAK2 and the STAT3 pathway, and TYK2 via the STAT4 pathway [12]. As many questions about safety and efficacy of small molecules still remain unclear, in our review we present the current data on approved JAK inhibitors, as well as those in clinical development for the treatment of IBD.

## 2. Mechanism of JAK Inhibitors Action

JAK family consists of four proteins: JAK1, JAK2, JAK3, and tyrosine kinase 2 (TYK2). They are intracellular tyrosine kinases that mediate the signal transduction via interaction with STATs pathway. Briefly, the ligand binding to the receptor causes its dimerization, leading to JAK phosphorylation and attraction of STATs to the docking sites. STATs also dimerize and relocate to the nucleus, resulting in gene transcription. JAK inhibitors are small molecules designed to interfere with the phosphorylation of JAK, suppressing the JAK-STAT downstream signaling. Importantly, different cytokines are bound to specific JAK receptors dimers, extending the therapeutic possibilities, as inhibiting a particular JAK is associated with a distinctive effect. The JAKs included in the transduction signal of chosen cytokines are depicted in Table 1. In case of IBD, many cytokines were shown to be primarily implicated in the pathogenesis and clinical course of the disease: IL-5, IL-9, IL-13 and IL-33 for UC, IL-10, IL-12, IL-27, and interferon (IFN)-γ, for CD and IL-6, IL-12, IL-17, IL-21, IL-23, and TNF-α for both [13,14]. Due to the different patient-to-patient specific cytokine profile, widening the target of action is advantageous compared to selective biologic agents. The JAK inhibitors are quickly absorbed to the bloodstream, have a low half-life time of about 3 h and are cleared mainly by the liver [15]. Noteworthy, they were shown to exert no immunogenicity. Thus, conversely to the therapies with biologic agents, the development of anti-drug-antibodies is not the issue during the JAK inhibitors treatment [16].

JAK inhibitors affect JAK isoforms with different selectivity, which seems to be relative and not absolute. JAK selectivity is also dose- and tissue-dependent. Some of the JAK inhibitors block all the JAKs with similar potency (non-selective or pan-selective): tofacitinib, peficitinib, or izencitinib (TD-1473), while others are more selective, such as filgotinib or upadacitinib. The selectivity of JAK inhibitors available on the market and in the developmental stage for the treatment of IBD is presented in Table 2. It is considered that higher selectivity is associated with less adverse events and less efficacy [11,17]. Ongoing trials will clarify whether the selectivity of these drugs has an impact on the safety and efficacy profile.

## 3. Non-Selective JAK Inhibitors

### 3.1. Tofacitinib (Pfizer)

Tofacitinib is the first JAKs inhibitor approved by FDA in 2018 for the treatment of patients with moderate-to-severe UC based on two induction and one 52-week maintenance randomized controlled trials (RTCs) [18]. Tofacitinib is a JAK1 and JAK3 inhibitor, hampering the downstream signaling of several cytokines (IL-6, IL-11, IFN-α/β, IFN-γ, and IL-10 through JAK1; IL-2, IL-4, IL-7, IL-9, IL-15, and IL-21 through JAK3) (Table 2) [18]. The results of OCTAVE Induction 1 and 2 trials [18] showed that remission at the 8th week was achieved by 18.5% and 16.6% of the patients with UC taking 10 mg of tofacitinib vs. 8.2% and 3.6% of the patients with UC in placebo group, respectively (*p* = 0.007 in OCTAVE Induction 1 and *p* < 0.001 in OCTAVE Induction 2). The remission was achieved by 34.3% of the patients taking 5 mg, while doubling the dose helped to maintain the remission in 40.6% of the patients compared to 11.1% with placebo (*p* < 0.001 for both groups). Results of the recent meta-analysis by Taconera et al. [19] showed that a remission was reached in as many as 47% of patients with UC at weeks 12–16, response in 64.2% at weeks 12–16, corticosteroid-free remission in 44.3% at weeks 12–16, and mucosal healing in 48.3% at week 8. These outcomes are in line with data gathered in clinical trials. A following dosing regimen is recommended by the National Institute of Health and Care Excellence for patients with UC: 10 mg twice daily for 8 weeks (induction phase) and 5 mg twice daily as a maintenance [20]. Accordingly, a recent RIVETING trial hinted that dose de-escalation from 10 mg to 5 mg twice daily in patients with UC with remission and anti-TNF-α naive, was safe [21].

Converse results were shown in three clinical trials addressing the potential use of tofacitinib in patients with CD. In a study conducted by Sandborn et al. [22], tofacitinib administered twice daily at doses of 1, 5, or 15 mg failed to achieve neither the significant clinical response nor the clinical remission during the 4-weeks long induction phase in patients with moderate to severe CD (NCT00615199). Consistently, outcomes of two randomized trials (NCT01393626 and NCT01393899) showed no significant efficacy of tofacitinib at the doses of 5 or 10 mg twice daily to achieve neither the clinical remission after the 8-weeks long induction phase nor the clinical response or remission at the end of the 26-weeks long maintenance phase [23]. Unexpectedly, a high proportion of patients with clinical response and remission in the placebo group may be one of the possible explanations of tofacitinib failure in the induction phase in the aforementioned studies. In this study, a greater proportion of patients was receiving corticosteroids, which are known to be effective in induction phase [23].

### 3.2. Peficitinib (ASP015K, Astellas Pharma)

Peficitinib is a non-selective JAK1/JAK3 inhibitor, which did not achieve statistically significant results in trial in patients with moderately to severely active UC [24]. The primary endpoint regarded as a dose-response to peficitinib at week 8 was not achieved. Clinical response was achieved by 34.1% of patients in 25 mg once daily (OD) group, 54.5% of patients in 75 mg OD group, 54.5% of patients in 150 mg OD group, and 54.5% of patients in the 75 mg twice daily (BID) group compared to 39.5% of patients in the placebo group. The clinical remission was achieved by 15.9%, 15.9%, 27.3% (*p* < 0.05), and 15.9% of patients, respectively, compared to 7% in the placebo group. Despite the evidence of one effective dose (150 mg, OD), more frequent adverse events were noted in patients taking doses of ≥75 mg [24]. No more trials regarding this molecule in IBD is active yet.

### 3.3. Izencitinib (TD-1473, Theravance Biopharm)

Targeting JAK/STAT signaling pathway, while offering promising treatment option for patients with IBD, is associated with some serious systemic adverse effect, especially in the case of non-selective JAK inhibitors [25]. Therefore, proof-of-concept has been provided for the potential therapeutic value of a gut-selective JAK inhibitor in UC patients, which leads to the development of TD-1473 molecule. In animal studies, where mice received orally 10 mg/kg of TD-1473 or tofacitinib, pharmacokinetic studies showed that systemic levels of TD-1473 were approximately 1000-fold lower than those of tofacitinib [26].

In a phase 1b of NCT02818686 study, TD-1473 in dose 20 mg OD (*n* = 10), 80 mg OD (*n* = 10), 270 mg (*n* = 10) OD or placebo (*n* = 9) was given to patients with moderately-to-severe active UC. Patients receiving the investigational drug more often experienced clinical response (50%, 20%, 20% vs. 11%, respectively) and endoscopic improvement (18%, 30%, 20% vs. 0%) in comparison to patients receiving placebo [26]. Interestingly, TD-1473 demonstrated approximately 40-fold stronger potency for TYK2 in comparison to tofacitinib [26]. As mentioned earlier, IL-23 and IL-12 are signaling via JAK2 and TYK2, but not JAK1 or JAK3 [27]. Thus, a pan-JAK inhibitor with higher potency for TYK2 might provide additional clinical benefits especially in CD, as seen for ustekinumab also targeting IL-12/IL-23 pathway [16].

Nowadays, Phase 2 and 3 trials in CD and UC are ongoing and to our knowledge no data is available. A phase 2 (NCT03635112; DIONE study), multi-center, randomized, double blind, placebo-controlled, parallel-group study evaluating the efficacy and safety of 12 weeks induction therapy with TD-1473 in subjects with moderate to severe active CD started in August 2018. According to the protocol, 160 patients are planned to be enrolled into the study and those who complete the induction phase will continue to receive TD-1473 for up to 48 additional weeks. Furthermore, a Phase 2b/3 (NCT03758443; RHEA study), randomized, double blinded, multi-dose, placebo-controlled studies was opened in November 2019 [28]. It consists of three separate studies, such as 8-week Phase 2b dose-finding induction study, an 8-week dose confirming Phase 3 induction study, and a 44-week Phase 3 maintenance study in patients with UC. Moreover, subjects, who have disease relapse or complete the maintenance study may be eligible to enter a separate long-term safety study.

## 4. Selective JAK Inhibitors

### 4.1. Upadacitinib (ABT-494, Abbvie)

Another designed JAK inhibitor, upadacitinib, selective to JAK1, was found to achieve clinical remission in patients with CD of moderate-to-severe activity. In CELEST study (NCT02365649) the clinical remission was confirmed in 11–27% patients receiving 3–24 mg of upadacitinib twice daily (*p* < 0.1 compared to placebo) and 14% of patients receiving 24 mg once daily compared to 11% patients in placebo group [29]. The rate of patients achieving clinical remission was not dose-dependent, meaning pharmacokinetics and pharmacodynamics can be unpredictable in CD. On the other hand, the statistical significance has been achieved in terms of the endoscopic remission. Depending on the used dose, 8–22% of patients taking upadacitinib achieved the endoscopic remission compared to 0% patients in placebo group (*p* < 0.05). More detailed studies in larger population are needed to exactly address the potency of upadacitinib in this entity.

In the U-ACHIEVE study (NCT02819635), addressing the potential of upadacitinib in patients with moderately to severely active UC, only the highest dose of the drug (45 mg) induced remission in a significant number of patients compared to placebo (19.6% vs. none, *p* = 0.002) [30]. Similarly to the CELEST study, upadacitinib was found more effective in inducing the endoscopic remission than achieving the primary outcome, described as a number of patients achieving clinical remission: 14.9%, 30.6%, 26.9%, and 35.7% of patients taking 7.5 mg, 15 mg, 30 mg, and 45 mg, resepctively achieved the endoscopic remission compared to 2.2% of patients in placebo group (*p* = 0.033, *p* < 0.001, *p* < 0.001, and *p* < 0.001, respectively).

### 4.2. Filgotinib (GLPG0634, Galapagos NV)

Filgotinib is another JAK1-selective inhibitor which rises as a plausible treatment option in patient with IBD. In the FITZROY study, filgotinib administered at the dose of 200 mg OD induced clinical remission in 47% of patients with moderate-to-severe CD compared with 23% in placebo group (*p* = 0.008) [31]. A clinical response was achieved in 59% of patients with CD vs. 41% of control group (*p* = 0.045). Of note, only 4 weeks were required for patients to achieve the clinical remission compared to tofacitinib, which showed a minimum period of 8 weeks to induce the remission. Few more studies analyzing the potential use of this JAK inhibitor in CD are ongoing: Divergence 1 (NCT03046056), addressing the potential use of filgotinib in the treatment of small bowel CD was completed, but no results are shown yet, Divergence 2 (NCT03077412), aiming at studying the efficacy of filgotinib in the treatment of perianal fistulizing CD is ongoing, Diversity1 (NCT02914561), which is recruiting patients to evaluate the safety and efficacy of filgotinib in moderate-to-severe CD, and DIVERSITYLTE (NCT02914600), a long-term extension study of Diversity1, used to assess the safety in patients with CD [32,33,34,35].

Only one trial evaluated the utility of filgotinib in UC—the SELECTION study (NCT02914522) [36]. It showed that a significantly higher portion of patients with moderate-to-severe UC, that was administered either 200 mg or 100 mg of filgotinib OD, achieved endoscopic/rectal bleeding/stool frequency remission compared to placebo group (37.2% vs. 11.2% and 23.8% vs. 13.5%, accordingly). Of note, the new indication for filgotinib in patients with moderately to severe UC is already under EMA evaluation.

### 4.3. SHR0302 (Reinstone Biopharma Company Limited)

SHR0302 is a novel, potent, and orally administered selective JAK1 inhibitor, with potentially a better safety and efficacy profile compared to the pan-JAK inhibitors. Due to JAK1 selectivity, SHR0302 use may cause fewer hematological side effects, which are related to JAK2 inhibition. Currently phase 2, randomized, double-blind, placebo-controlled, four arm parallel, multi-center studies are ongoing to investigate the safety and efficacy of SHR0302 in patients with moderate to severe active UC (NCT03675477; AMBER2) and CD (NCT03677648) [37,38].

The manufacturer announced on 4 February 2021, that SHR0302 met the primary and secondary endpoints in AMBER2 (NCT03675477) study, where SHR0302 at the doses of 8 mg OD, 4 mg BID, and 4 mg OD in 164 adults with moderate to severe UC was investigated [39]. The AMBER2 study consisted of an 8-week induction period, followed by an 8-week extension period. Statistically significant clinical response at week 8 was achieved in all active arms: 8 mg OD (46.3%), 4 mg BID (46.3%), and 4 mg OD (43.9%) in comparison to placebo (26.8%). Furthermore, administration of SHR0302 resulted in a higher percentage of patients who achieved clinical remission in all treatment arms compared to placebo [39]. NCT03677648 is a Phase 2 study with 12 weeks treatment phase and 12-week extension phase, where 144 participants are planned to be recruited. The estimated study completion date is October 2021 and to our knowledge, currently, there is no data about the safety and efficacy of SHR0302 in CD [38].

### 4.4. OST-122 (Oncostellae)

According to the manufacturer, OST-122 is an oral, gut-restricted, and subtype-selective JAK3/TYK2/AMPK-related protein kinase 5 (ARK5) inhibitor for the local treatment of IBD including UC, CD and, potentially, fibrotic lesions in Crohn’s patients [38]. The compound was well tolerated in a Phase 1 study in healthy volunteers and has been shown to be stable during the GI transit, while no significant plasma levels were detected. After multiple dosing, minimal plasma levels have been measured with individual C_max_ values below 3.5 ng/mL. Thus, the gut-restricted pharmacokinetic (PK) profile of OST-122 lowers the risk of systemic toxicities inherent to other JAK inhibitors [40].

Phase 1b/2a (NCT04353791), a randomized, double-blinded, placebo-controlled, multi-center clinical trial used to investigate the safety, pharmacokinetics, and efficacy of oral treatment with OST-122 for 28 days in 32 patients with moderate to severe UC was started in 2020 [40]. Estimated trial completion date is April 2022 and no data about its outcome is currently available.

### 4.5. Deucravacitinib (BMS-986165, Bristol-Myers Squibb)

BMS-986165 is a novel, oral selective TYK2 inhibitor, which targets IL-12, Il-23, and type 1 IFN. BMS-986165 binds to the unique regulatory domain of TYK2 and specifically inhibits TYK2 signaling, unlike JAK1–3 inhibitors, which binds to the active domain adenosine triphosphate (ATP) site common to all JAK molecules [41,42]. Deucravacitinib was already found effective in treating psoriasis [43], and nowadays is still in trials in the IBD field. A Phase 2, randomized, double blinded, placebo controlled clinical trial (NCT03599622; LATTICE study) to investigate the efficacy and safety of BMS-986165 in patients with moderate to severe CD was launched in 2018. Its main goal is to enroll 240 patients with CD and evaluate 2 doses of BMS-986165 [44]. Furthermore, a parallel Phase 2, randomized, double-blinded, placebo-controlled study (NCT03934216; LATTICE-UC) to evaluate the efficacy and safety of BMS-986165 in patients with moderate to severe ulcerative colitis started in April 2019 [45]. According to the available sponsor’s information, 120 patients are going to be enrolled and the study completion date is August 2021. Currently, both studies still have a recruiting status and no results are available.

### 4.6. Other JAK Inhibitors

There is a wide range of new JAK inhibitors being under development not only in IBD but also in other inflammatory conditions. The role of these novel compounds as the first line treatment, as well as in combination with other molecules with different mechanism of action for the treatment of IBD needs further clarification. Ritlecitinib (PF-06651600) is a dual irreversible inhibitor of JAK3 and the tyrosine kinase expressed in hepatocellular carcinoma (TEC) kinase family. The addition of a TEC inhibitor widens the anti-inflammatory potential and some TEC family members are considered as a treatment target in inflammatory diseases [46].

Another small molecule, brepocitinib (PF-6700841) is designed as a dual inhibitor of TYK2 and JAK1 [47]. Additionally, to the modulation of cytokine spectrum covered by the latter, the former regulates the IL-12, IL-23, and Type 1 IFN signaling.

Currently, two trials are listed, comparing these agents in two arms vs. placebo in CD (NCT03395184) and UC (NCT02958865). Both agents appeared to be effective in alleviating the inflammation in RA [47]. Whether they will serve as another treatment option in IBD is yet to be clarified.

The group of available JAK inhibitors is complemented by itacitinib (INCB039110), solcitinib (GSK2586184), delgocitinib (JTE-052), baricitinib (INCB28050, LY3009104), ruxotinib (INCB018424, INC424), oclacitinib (PF-03394197), abrocitinib (PF-04965842), and decernotinib (VX-509). However, the majority of these JAK inhibitors were not investigated as a potential therapeutical option in IBD. Only solcitinib was evaluated in patients with UC (NCT02000453), but the study was terminated due to the safety concerns that rose in a parallel study with systemic lupus erythematous.

Another small molecule is baricitinib, a selective inhibitor of JAK1 and JAK2, with less potency for JAK3. It has received regulatory approval from FDA and EMA for the treatment of moderately to severely active RA [48]. There are no clinical trials including baricitinib as an IBD therapy, however, the multipotential activity of this drug, especially its antiviral effect, makes it an interesting therapeutic option in the time of the coronavirus disease-2019 (COVID-19) pandemic [48]. Another small molecule, itacitinib is an orally bioavailable inhibitor of JAK1 with potential antineoplastic and immunomodulating activities. Specifically, itacitinib is 20 times more selective for JAK1 than for JAK2 and 200 times more than for JAK3 [49]. To date, itacitinib has been used in trials studying the treatment of chronic graft-versus-host-disease, RA, and advanced solid tumors, such as pancreatic cancer [25,50,51]. In the 2018 Phase 2 study, a double-blind, dose-ranging, placebo-controlled study with open-label extension to evaluate the efficacy and safety of itacitinib in patients with moderate to severe UC was started (NCT03627052) [52]. However, due to the lack of participants study it was closed in 2019 and no data about itacitinib treatment in IBD was presented. The major RCTs are listed in Table 3.

### 4.7. Safety of JAK Inhibitors

**Figure 1:**
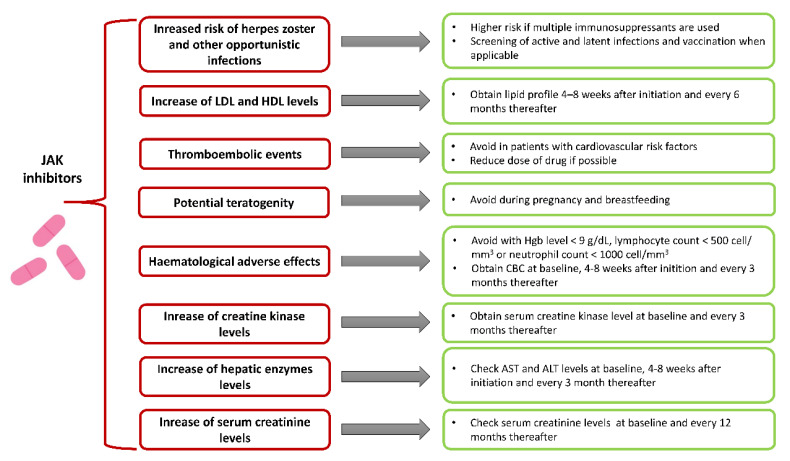
Management of potential AE associated with JAK inhibition. JAK-associated receptors are widely distributed in the human body and possess numerous and varied functions. Thus, appropriate monitoring is needed in patients treated with JAK inhibitors to minimalize AE associated with JAK inhibitors. Potential adverse effects and strategies for its prevention and monitoring, when applicable, are presented in this Figure. Currently, only one JAK inhibitor, tofacitinib, is available on the market, hence recommendations are based on the prescribed information for it and expert opinions. AE, adverse effect; ALT, alanine transaminase; AST, aspartate transaminase.

A great number of growth factors, hormones, and cytokine receptors act through JAKs and thus, JAK functions may be considered as a pleiotropic. Although JAK inhibitors are developed to block specific JAKs, higher doses may have effect on multiple JAKs, causing off-target binding and leading to immunosuppressive, metabolic, hepatotoxic, and hematological effects (Figure 1) [42]. Moreover, as every drug, JAK inhibitors could induce drug allergies and induce drug-drug interactions. Nevertheless, these potential risks, evidenced from RCTs involving patients with IBD, rheumatoid arthritis, and psoriasis, support a favorable safety profile for these agents [12].

The clinical studies with tofacitinib showed no overall difference in adverse events (AE) between tofacitinib in dose 10 mg twice daily and placebo [18]. The most frequent reported AE were nasopharyngitis, influenza, arthralgia, and headache. Importantly, in OCTAVE induction studies, the infection rates were more common in patients treated with tofacitinib compared to the placebo group. Moreover, serious infections were more frequently observed in patients receiving tofacitinib (1.3% vs. 0% in OCTAVE I and 0.2% vs. 0 in OCTAVE 2) [18]. Results from the OCTAVE Sustain study showed that tofacitinib in dose 10 mg is associated with higher rates of *Herpes zoster* infection (5.1%) than tofacitinib in dose 5 mg (1.5%) and placebo (0.5%), although no serious cases have been observed [18]. This study revealed several risk factors of *Herpes zoster* infection, such as age >65 years, previous TNF inhibitor failure, and Asian ethnicity [53]. Further integrated analysis confirmed the dose-dependent risk of *Herpes zoster* infection in patients with ulcerative colitis, but other opportunistic infections were infrequent [54]. Furthermore, a similar AE profile has been demonstrated in long-term data with patients with rheumatoid arthritis receiving tofacitinib over 8.5 years [55]. According to data gathered from studies with patients with RA and pooled analysis of ulcerative colitis, studies showed that risk of lymphoma and other malignancies were low [12,54].

Of note, post-marketing reports from studies in patients with RA raised concerns that treatment with JAK inhibitors may be associated with a higher risk of thrombotic events [56]. Contrary, some analyses have shown that these events are uncommon and may be related to other patients’ diseases [57,58]. Nevertheless, FDA released two box warnings for increased risk of pulmonary embolism and mortality with a 10 mg dose of tofacitinib BID [59]. FDA recommendations updated the prescribing information, stating that tofacitinib should be used at the lowest dose for the shortest duration possible to achieve and maintain a therapeutic response. Moreover, current recommendations suggest that maintenance therapy dosing should not exceed 5 or 10 mg BID.

As mentioned earlier, JAKs plays an important role in hematopoiesis, thus laboratory parameters should be periodically assessed to actively search for AE [54]. Moreover, elevated levels of serum liver transaminases, creatinine, and creatine phosphokinase have been reported, but no relation between these elevations and liver or renal failure as well as rhabdomyolysis were observed [53,54,60]. A dose-dependent increase in low-density lipoprotein (LDL) and high density lipoprotein (HDL)-cholesterol levels has also been observed, however this finding was not associated with a higher incidence of cardiovascular event in patients with RA followed up for 8.5 years [55]. In the study performed by Sands et al. [24], the evaluation efficiency of peficitinib in treatment of moderate-to-severe ulcerative colitis patients receiving dose of peficitinib > 75 mg per day had more frequently elevated fasting lipids levels, creatine phosphokinase levels compared to placebo group. Importantly, all changes in laboratory parameters described earlier are reversible after the therapy cessation. Currently, the safety of JAK inhibitors during pregnancy and breast feeding has not been well established in larger cohorts. However, tofacitinib have exhibited teratogenic and feticidal properties in animal models when used at doses several times higher than those used in humans [57]. Based on this information, JAK inhibitors should be avoided in this population.

To date, in 5 tofacitinib trails in patients with UC, only 11 subjects were diagnosed with a malignancy excluding non-melanoma skin cancer. All of them previously received treatment with thiopurines or TNF inhibitors. All reported malignances were discrete and included cervical cancer, hepatic angiosarcoma, cholangiocarcinoma, cutaneous leiomyosarcoma, Epstein-Barr virus-associated lymphoma, renal cell carcinoma, acute myeloid leukemia, adenocarcinoma of colon, lung cancer, and breast cancer. Moreover, 11 patients with non-melanoma skin cancer were reported. The incidence rates of malignancy were 0.7 (95% CI, 0.3–1.2) per 100 patients of exposure. Currently, there is not enough data to determine cancer risk due to tofacitinib and other JAK inhibitors. In the absence of clear evidence, it is reasonable to avoid JAK inhibitors or use them with caution among patients with history of cancer.

Several next-generation JAK inhibitors are currently in clinical development (Table 2 and Table 3). Those agents, due to greater selectivity for specific JAKs (filgotinib, upadacitinib) or gut selective drugs (TD-1473), may have an improved safety profile. Data from a Phase 2 study of patients with CD treated with filgotinib, the JAK1-selective inhibitor, showed no differences in serious AE compared with the placebo. Nevertheless, serious infections were observed in 3% of patients from filgotinib group compared to none in the placebo group [24]. In another study comparing different doses of upadacitinib in CD patients, no dose-dependent rates of AE were found, however, the study lacked a placebo group [61]. Another 28-day phase Ib study evaluated the safety of the gut-selective pan-JAK inhibitor TD-1473 in 40 patients with UC. Importantly, no cases of serious or opportunistic infections were reported [28].

Available data suggest that JAK inhibitors, especially the new generation, may be useful in IBD therapy. Nevertheless, the complexity of JAK-STAT pathways and the potential for uncommon reactions suggest that safety evaluation of these agent should be investigated in large clinical studies as well as appropriate post-marketing studies.

### 4.8. JAK Inhibitors in IBD in the Light of COVID-19 Pandemic

Currently, the available data according to the safety of JAK inhibitors use in patients with IBD during the COVID-19 pandemic are limited. It is known that the use of immunosuppressants during an acute viral disease may carry the risk of delayed virus clearance and increased susceptibility to secondary opportunistic infections [62,63]. Previously, at the beginning of the COVID-19 pandemic, patients with IBD treated with immunomodulators were considered to be more vulnerable to severe acute respiratory syndrome coronavirus-2 (SARS-CoV-2) infection and to have a higher risk of more severe course of COVID-19. For this reason, the Chinese Society of Gastroenterology, due to rapidly increasing number of SARS-CoV-2 infection cases, recommended withholding immunotherapy [64,65]. However, according to the current evidence, it is suggested that IBD patients on immunotherapy, apart from those treated with corticosteroids, do not seem to have an increased risk of developing COVID-19 or a severe infection outcome [66,67,68,69,70]. Currently available studies have shown that predictors associated with a higher risk of COVID-19 and related severe course or death among patients with IBD are older age, active disease, and presence of comorbidities [66,68,70]. According to recently published recommendations from different gastroenterological societies, such as the British Society of Gastroenterology (BSG), European Crohn’s and Colitis Organization (ECCO) and International Organization for the Study of Inflammatory Bowel Diseases (IOIBD), it is advisable to maintain immunotherapy, apart from corticosteroids, in order to avoid relapse [64,71,72,73]. Furthermore, relapse of IBD may require steroid therapy or hospitalization, which are both risk factors of COVID-19 [72]. All of the three mentioned societies also recommend continuation of JAK inhibitors treatment. In case SARS-CoV-2 infection develops, ECCO and IOIBD suggest withholding JAK inhibitors until resolution. Due to some indication that lower T-helper lymphocyte counts are associated with delayed clearance of viral RNA, ECCO recommends against initiation of tofacitinib if therapeutic alternatives are available, in contrast to BSG and IOIBD [74].

The Surveillance Epidemiology of Coronavirus Under Research Exclusion for Inflammatory Bowel Disease (SECURE-IBD) database, (www.covidibd.org (accessed on 21 October 2021)) represents the reliable source of evidence about outcomes of IBD patients with SARS-CoV-2 infection, including the impact of immunosuppressive therapy. It is an international, collaborative registry of adult and pediatric IBD patients, established in March 2020. It also provides a free online risk calculator for physicians, which can serve as a prognostic tool of COVID-19- related adverse outcomes in a population of IBD patients, distinguishing between high- and low-risk patients to adjust personalized clinical guidance [75]. Based on SECURE-IBD registry, according to data reported between March and September 2020, only 1.6% of IBD patients (37 of 2326) were treated with tofacitinib [76]. There were no significant differences in COVID-19 outcomes, including the occurrence of hospitalization (21.6% vs. 23.3%), admission to the Intensive Care Unit (ICU) (5.4% vs. 4.5%) or severe infection course (6.2% in both groups), comparing tofacitinib and other medications-treated IBD patients [76]. Some concerns regarding tofacitinib use during SARS-CoV-2 infection may also arise due to association of this drug with increased risk of infectious viral complications, particularly *Herpes zoster* infection [18]. However, more evidence is needed to assess the risk of tofacitinib use in other viral infections, including COVID-19.

Moreover, one of the main concerns about using JAK inhibitors in COVID-19 is the risk of thromboembolic events. Patients with COVID-19 appear to have an increased susceptibility for immunothrombosis, which is noticed in up to one third of patients with severe SARS-CoV-2 infection [77,78]. An increased incidence of thromboembolic events was reported with higher doses of tofaticinib, as well [79]. Thrombotic complications may become a serious limitation of JAK inhibitors use in SARS-CoV2 infection, however, while waiting for larger studies, these early data should be treated cautiously.

Other data suggest that some JAK inhibitors may even have a potential therapeutic role in the management of COVID-19, according to the theory that a hyperinflammatory response to SARS-CoV-2 infection with an excessive release of cytokines is involved in the underlying pathophysiology of acute respiratory distress syndrome (ARDS) and multi-organ failure [80,81,82]. Baricitinib is under investigation in multiple ongoing clinical studies, being considered for the treatment of COVID-19 with hyperinflammation and ARDS, due to its dual anti-inflammatory effect and potential antiviral activity [82,83,84]. It can moderate the cytokine storm (including IL-6 and IFN-γ) and inhibits Numb-Associated Kinase, which is involved in SARS-CoV-2 cell entry [82,83]. Tofacitinib, unlike baricitinib, has not shown detectable inhibition of NAK [82]. Further randomized studies may clarify all the unanswered questions. Currently, managing IBD patients during the COVID-19 pandemic, we are still dependent mostly on anecdotal evidence, expert opinions, and societies guidelines.

## 5. Conclusions

Targeting the JAK-STAT pathway and multiple pro-inflammatory cytokines seems to have a great potential for novel IBD treatment, especially in the case of loss of response to another therapy. Several JAK inhibitors have shown promising results for induction and maintenance of remission in moderate to severe UC or CD. This drug class may become a convenient alternative for biologic therapies, due to oral administration, rapid onset of action, or lack of immunogenicity. Multiple questions remain regarding JAK inhibitors safety profile, including the risk of opportunistic infections, thromboembolic events, or long-term effects. Large, longitudinal studies are needed to clarify the risk-benefit ratio of these novel drugs and to determine place of JAK inhibitors in the management of IBD.

## Figures and Tables

**Table 1:** Cytokines and growth factors targeted by specific JAKs.

JAK1	JAK2	JAK3	TYK2
IL-2	IL-3	IL-2	IL-6
IL-4	IL-5	IL-4	IL-11
IL-6	IL-6	IL-7	IL-12
IL-7	IL-11	IL-9	IL-23
IL-9	IL-12	IL-15	Oncostatin M
IL-10	IL-23	IL-21	Leukemia inhibitory factor
IL-11	Oncostatin M		IFN-α/β
IL-15	Leukemia inhibitory factor		
Oncostatin M	Granulocyte-macrophage colony stimulating factor		
Leukemia inhibitory factor	Erythropoietin		
IFN-α/β	Thrombopoietin		
IFN-γ	IFN-γ		

**Table 2:** Selectivity of Jak inhibitors available on market and in the development stage for the treatment of IBD.

JAK-Associated Receptors	Non-Selective or Pan-Selective			Selective					
	Tofacitinib	Peficitinib (ASP015K)	(Izencitinib) TD-1473	Filgotinib (JAK1)	Upadacitinib (JAK1)	SHR0302 (JAK1)	Itacitinib (JAK1)	OST-122 (JAK3/TYK2/ARK5)	BMS-986165 (TYK2)
**Type I cytokine receptor**									
JAK1, JAK2, TYK2	+	+	+	+	+	+	+	+	+
JAK 1, JAK3,	+	+	+	+	+	+	+	+	-
JAK2, TYK2	+	+	+	-	-	-	-	+	+
JAK-2	+	+	+	-	-	-	-	-	-
**Type II cytokine receptor**									
JAK1, JAK2, TYK2	+	+	+	+	+	+	+	+	+
JAK1, JAK2	+	+	+	+	+	+	+	-	-
JAK1, TYK2	+	+	+	+	+	+	+	+	+

“-” no inhibitory effect, “+” inhibitory effect.

**Table 3:** Major RCTs regarding efficacy of JAK inhibitors in IBD.

RCT Identifier	Drug Studied	JAK	Disease	Dose
NCT01465763 [18]	Tofacitinib	JAK1/JAK3	UC	10 mg, BID
NCT01458951 [18]				
NCT03281304 [21]				10 mg→5 mg, BID
NCT00615199 [22]			CD	1, 5, 15 mg, BID
NCT01393626 [23]				5, 10 mg, BID
NCT01959282 [24]	Peficitinib	JAK1/JAK3	UC	25, 75, 150 mg, OD 75 mg BID
NCT02818686 [26]	Izencitinib	pan-JAK	UC	20, 80, 270 mg, OD
NCT03635112			CD	N/A
NCT03758443 [28]			UC	N/A
NCT02365649 [29]	Upadacitinib	JAK1	CD	3, 6, 12, 24 mg, BID 24 mg, OD
NCT02819635 [30]			UC	7.5, 15, 30, 45 mg, OD
NCT02048618 [31]	Filgotinib	JAK1	CD	200 mg, OD
NCT03046056 [32]				N/A
NCT03077412 [33]				
NCT02914561 [34]				
NCT02914600 [35]				
NCT02914522 [36]			UC	100, 200 mg, OD
NCT03675477 [37,39]	SHR0302	JAK1	UC	4, 8 mg, OD 4 mg, BID
NCT03677648 [38]			CD	N/A
NCT04353791 [40]	OST-122	JAK3/TYK2/ARK5	UC	N/A
NCT03599622 [44]	Deucravacitinib	TYK2	CD	N/A
NCT03934216 [45]			UC	N/A
NCT03395184	Ritlecitinib	JAK3/TEC	CD	30, 60 mg, OD
NCT02958865			UC	
NCT03395184	Brepocitinib	JAK1/TYK2	CD	50, 200 mg, OD
NCT02958865			UC	

## Abbreviations (in Alphabetical Order)

AE	Adverse event
ARK5	AMPK-related protein kinase 5
ARDS	Acute respiratory distress syndrome
ATP	Adenosine triphosphate
BID	Twice daily
BSG	British Society of Gastroenterology
CD	Crohn’s disease
COVID-19	Coronavirus disease 2019
ECCO	European Crohn’s and Colitis Organization
EMA	European Medicines Agency
FDA	Food and Drug Administration
GI	Gastrointestinal
HDL	High density lipoprotein
IBD	Inflammatory bowel disease
IFN	Interferon
IOIBD	International Organization for the Study of Inflammatory Bowel Diseases
JAK	Janus kinase
LDL	Low-density lipoprotein
OD	Once daily
RA	Rheumatoid arthritis
RCT	Randomized controlled trial
SARS-CoV-2	Severe Acute Respiratory Syndrome Coronavirus 2
STATs	Signal transducers and activators of transcription
TNF-α	Tumor necrosis factor α
TYK2	Tyrosine kinase 2
UC	Ulcerative colitis
